# Numerical, Physical, and Industrial Investigations on Hot Metal Desulphurization—From Macromixing Conditions to Reaction Rate Phenomena

**DOI:** 10.3390/ma17235858

**Published:** 2024-11-29

**Authors:** Adam Cwudziński, Jan Falkus, Angelika Podolska-Loska

**Affiliations:** 1 Department of Metallurgy and Metals Technology, Faculty of Production Engineering and Materials Technology, Czestochowa University of Technology, Armii Krajowej 19, 42-201 Czestochowa, Poland; 2 Department of Metal Forming and Metallurgical Engineering, Faculty of Metals Engineering and Industrial Computer Science, AGH University of Krakow, Adama Mickiewicza 30 Ave., 30-059 Kraków, Poland; jfalkus@agh.edu.pl (J.F.); podolska@agh.edu.pl (A.P.-L.); 3 ArcelorMittal Poland S.A., Józefa Piłsudskiego 92 Ave., 41-308 Dabrowa Gornicza, Poland

**Keywords:** hot metal, sulphur removal, mixing phenomena, ladle, numerical and physical modeling, industrial trials

## Abstract

The efficiency of the hot metal pretreatment process plays a very important role in achieving high-quality and low-cost advanced steel. From macromixing phenomena obtained by numerical modeling and physical experiments to compound interaction databases from industry trials, the Authors compared fundamental relationships from the literature with their own laboratory results and plant data. A simple numerical model based on the LES turbulence approach was well-validated by water modeling. Hence for a 300-ton ladle, the mixing time and mixing power were predicted. Finally, the mass transfer controlled rate was calculated based on a computational fluid dynamics model and thermodynamic model, which indicated results limitations. Moreover, from the industry data, it was found that the rate constant of the desulfurization process varies within a wide range, affecting the efficiency of the sulphur removal degree from values 0.6 to 0.98.

## 1. Introduction

According to the guidelines of the European Commission, the iron and steel industry should intensify their reduction in CO_2_ emissions. In steelworks, with a full production cycle implementing steel-melting technology based on natural raw materials, the production of sinter, coke, and hot metal is an important source of CO_2_ emissions. Hence, there is a need to optimize the existing technologies or implement new ones based on blue or green hydrogen. One of the technological areas in which optimization can be implemented is the pretreatment of blast furnace iron, during which the sulfur content in the hot metal is reduced before the basic oxygen furnace (BOF) steelmaking process. Based on the chemical affinity of sulfur for calcium and magnesium, appropriate mixtures are designed and fed in a nitrogen gas stream to the liquid hot metal through an injection top lance. Chemical reactions between sulfur in the hot metal and the desulfurizing agent take place in the volume of the liquid metal and at the metal–slag interface. However, vigorous mixing of the system, which ensures a developed contact boundary between the reactants, is the basis of the desulfurization process, stimulating the rate of transfer of substrates in coexisting phases. From a fundamental point of view, the submerged powder injection process is controlled via the reaction rate, mixing rate, and feeding rate [1]. The parameters influencing the mixing and desulfurization process are the volumetric flow rate of the gas stream as a carrier of the desulfurization agent and a momentum stimulator in the system (mixing power), correlating with the parameter of penetration of the desulfurization agent into the volume of hot metal [2,3,4], the shape of the injection lance head—including the number of injection holes, number of lances, and the position of the top lance in relation to the axis of the ladle [5,6,7,8]—type of desulfurizing agent (chemical composition and size) [9,10,11,12,13,14], or treatment time [15,16]. Due to top lance injection process, slopping, splashing phenomena, and swirl motions are expected [17,18,19]. From a fluid mechanics point of view (a strong stirring mechanism), the hot metal pretreatment process can be developed by scaling results from other metallurgical reactors such as top-submerged-lance furnaces, basic oxygen furnaces, or RH tank degassing ladles [20,21,22,23,24,25]. Reducing the sulfur content before the BOF process allows for a reduction in the amount of slag-forming materials introduced into the oxygen converter, stabilizing steel smelting by reducing the consumption of scrap and refractory lining. The fundamental knowledge concludes that the desulphurization process depends simultaneously on the stirring rate of hot metal and the sulphur distribution ratio, i.e., the equilibrium sulfur content of the hot metal and sulfur content of the slag/desulphurization agent [26]. Modeling of mixing phenomena was developed over 30 years by optimizing different mathematical turbulence models and observing liquid–gas systems in the water model [27,28,29,30,31,32]. From the previous work, the Authors found that the kinetics of injection processes depend on the carrier gas, gas flow rate, particle size, solid flow rate, lance diameter, surface tension, bath height, injector positions, or system equilibrium [33,34,35,36,37,38,39,40,41,42,43,44,45].

The information presented in the literature on mathematical models allows for the prediction of the best technology conditions for increasing the yields of the desulphurization process and decreasing the process costs. Barron et al., via mathematical simulation, estimated that the top lance process can be more sufficient than the process with rotary impellers [46]. Vuolio et al. used a genetic algorithm to predict the interaction of the desulfurization agent with hot metal [47]; from the obtained results, the Authors conclude that the main factor for process efficiency is the amount of used agent. At the same time, the data indicate that the flow rate of an agent and the activities of hot metal components have a minor effect on the process. Meanwhile, Pirker et al. used computational fluid models to predict the possibility of using a top lance in slag phase regeneration during hot metal desulphurization; the Authors found that dipper immersion of the top lance increases process efficiency [48]. Moosavi-Khoonsari et al. investigated the hot metal desulphurization process by using a mathematical kinetics model. This model predicted treatment efficiency very well depending on the type and amount of agent injection to hot metal [49]. In addition, a water model experiment allowed metallurgical engineers to develop technologies for hot metal desulphurization. Torres et al. tested a system agitated by bottom gas injection and impellers with injection nozzles [50]; the Authors conclude that the desulphurization process can be effectively improved by gas injection, which stimulates slag dispersion, and flux dispersion, which improves the mass transfer rate. In the work realized by Liu et al., a cold model allowed for choosing the best design of an impeller to process bubble dispersion and disintegration in the bulk of hot metal [51]. Finally, the Authors strongly agree with Deo et al.—only when several paths of the process are recognized and considered, and suitable results are compared and analyzed from laboratory trials, mathematical modeling, and industry, will full technological success be possible [52].

The present work describes the mixing phenomenon of hot metal and correlates the obtained hydrodynamic results with the mass transfer mechanism of sulfur from the hot metal volume to the desulphurization agents and forming slag. The Authors confirm that a simple numerical model composed of a large-eddy simulation (LES) turbulence approach with a discrete phase model—which describes the gas phase—and with a flat top liquid steel surface without additional user-defined functions adequately describes a strongly agitated system. The correctness of the proposed mathematical approach was validated by the Authors’ own water model investigations and the empirical formula proposed by Mazumdar and Guthrie. Moreover, the Authors analyzed the process of hot metal desulphurization based on industry data, and the thermochemistry calculations showed and confirmed that the factors that limit hot metal pretreatment processes are not only mixing but also reaction and feeding rate phenomena.

## 2. Ladle Description

**Figure 1:**
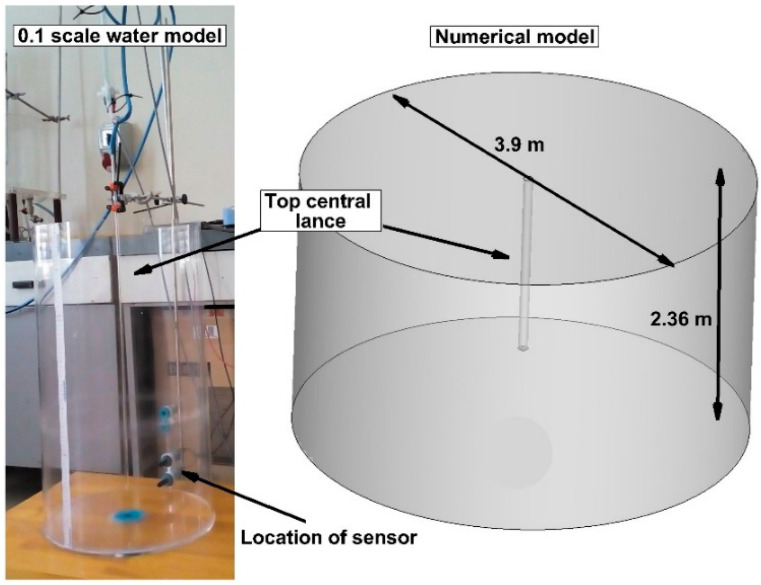
Sketch of the numerical model of Ladle No. 1 and the 0.1-scale water model of Ladle No. 1.

For the treatment of hot metal before the BOF process, the hot metal in the ladle is transferred to a station for blowing with desulfurizing powders. In order to assess the hydrodynamic conditions and mixing potential as well as the kinetics of the desulfurization process, two different capacities of the ladle were considered. For a ladle capacity of 201 tons (Ladle No. 1), a Plexiglas physical model was made on a linear scale of 0.1 (Figure 1).

**Figure 2:**
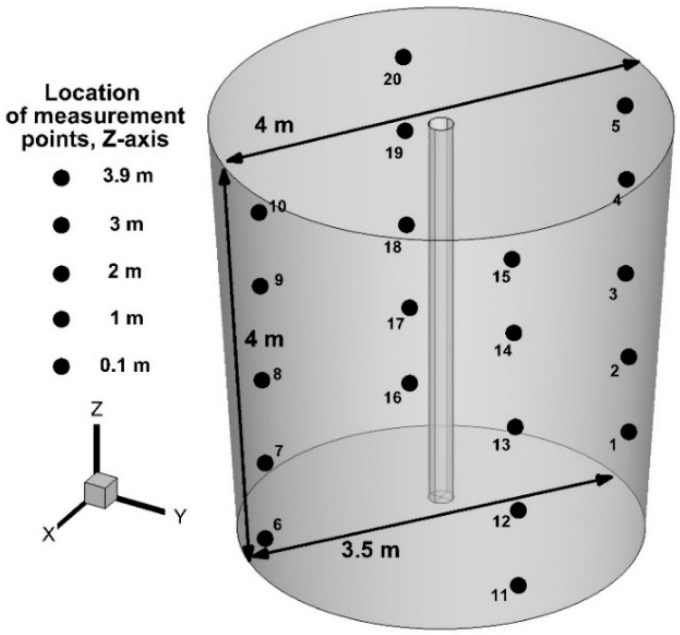
Industry 315-ton ladle (Ladle No. 2) numerical model with locations of monitoring points.

The base diameter of the physical model was 0.39 m. The physical model was filled with water to a height of 0.236 m. The internal diameter of the top injection lance in the physical model was 0.004 m, and the lance wall thickness was 0.002 m. The distance of the top injection lance from the bottom of the physical model was equal to 0.07 m. The second numerical model of the ladle (Ladle No. 2), with a truncated cone shape and a nominal capacity of 315 tons, was close to industry devices. In Ladle No. 2, the internal diameter of the top injection lance was 0.026 m and its external diameter was 0.22 m (Figure 2).

The distance from the bottom of Ladle No. 2 to the lance tip was 0.3 m. Both numerical ladle models did not include the freeboard volume necessary in an industrial process.

## 3. Methodology

The basic similarity criterion used in the tests was the Froude criterion, in accordance with which, the nitrogen flow rate and the correlation of the liquid hot metal treatment time between the physical model and the numerical model were calculated. The methodology used for similarity criteria is valid for adequately reflecting mixing phenomena inside hot metal bulk created by gas plumes and tested successfully in the Authors’ previous work [44,45,46,47,48,49,53,54,55,56,57,58].

(1) $$Q_{m}=\lambda^{2.5}Q_{il}$$

(2) $$t_{m}=\sqrt{\lambda }t_{it}$$

where *Q_m_*—volumetric gas flow rate in the physical model; *Q_il_*—volumetric gas flow rate in the industry ladle; *t_m_*—time of physical trials; *t_it_*—time of industry treatment; *λ*—scale factor.

In the physical modeling, the liquid imitating liquid hot metal was distilled water. Nitrogen was blown through the top central injection lance at a flow rate of 7 NL/min. In order to determine the mixing characteristics, 10 mL of 10% KOH solution was introduced through the injection lance. It should be noted that the amount and concentration of tracer used during the physical experiment influences the obtained results [59,60,61]. Therefore, for the possibility of comparing results between two considered systems, water–nitrogen and hot metal–nitrogen, the Authors maintained a similarity of the tracers used for both systems. At a distance of 0.07 m from the bottom of the model, in the side wall, there was a conductometric sensor for measuring the specific conductivity of water, enabling conductivity sampling at a frequency of 1 Hz. The physical stand and methodology of the experiments are clearly described in the Author’s previous work, [62]. The physical experiment was repeated eight times to obtain a reliable picture of the mixing process. A monitoring point for tracer concentration changes was designated in the volume of the numerical model (Ladle No. 1), corresponding to the location of the conductometric sensor in the physical model (Figure 1), in which tracer concentration changes were analyzed at a frequency of 2 Hz.

Since the mixing rate varies in individual zones of the working volume of batch reactors, such as ladles, especially in the wall areas, 20 monitoring points were designated in the mathematical model, reflecting Ladle No. 2 where the change in tracer concentration was recorded as a function of time (Figure 2). Monitoring points were located 1.65 m from the central axis of the ladle toward the side wall. Additionally, the points were located at five height levels, i.e., 0.1, 1, 2, 3, and 3.9 m from the bottom of the ladle. For modeling high-temperature system computer fluid dynamics (CFD), conception was used. Hence, computer simulations of the behavior of hot metal in both ladles during the injection of the nitrogen were performed in the Ansys-Fluent 12.1 program. The following properties for hot metal during numerical simulations were selected: *ρ*—7149 kg/m^3^, µ—0.00794 Pa s, Cp—750 J/kg K [63], and k_T_—41 W/m K [63]. The values of hot metal density and viscosity were determined from empirical formulas [63,64]. The density of nitrogen at a standard state was 1.138 kg/m^3^ (numerical simulations for Ladle No. 1). This value was taken from the Ansys-Fluent 12.1 database. Due to a low depth level of the water and the water room-temperature, expansion of nitrogen during physical trials was insignificant. Therefore, the density of nitrogen at a standard state was 1.138 kg/m^3^ and was considered during numerical simulations for Ladle No. 1. The large-eddy simulation (LES) model was used to describe the turbulence in the system, in which the instability of the turbulence of large eddies is calculated directly, while the turbulence at the level of small eddies is modeled using a subgrid-scale model. For an incompressible Newtonian fluid, the continuity and momentum equations take the following form:

(3) $$\frac{\partial {\bar{u}}_{i}}{\partial {\bar{x}}_{i}}=0$$

(4) $$\frac{\partial {\bar{u}}_{i}}{\partial t}+\frac{\partial \left({\bar{u}}_{i}{\bar{u}}_{j}\right)}{\partial x_{j}}=-\frac{1}{\rho }\frac{\partial \bar{p}}{\partial x_{i}}-\frac{\partial \tau_{ij}^{r}}{\partial x_{j}}+\nu \frac{\partial^{2}{\bar{u}}_{i}}{\partial x_{i}\partial x_{j}}$$

(5) $$\tau_{ij}^{r}=-2\nu_{t}\left(\frac{1}{2}\left(\frac{\partial {\bar{u}}_{i}}{\partial x_{j}}+\frac{\partial {\bar{u}}_{j}}{\partial x_{i}}\right)\right)$$

where *u*—velocity; *p*—pressure; *ρ*—density; *ν*—kinematic viscosity; *ν_t_*—eddy kinematic viscosity; *τ*—stress tensor; *t*—time.

The subgrid-scale model used in the numerical simulations was the kinetic-energy transport model, where kinetic energy and eddy kinematic viscosity are presented by the following formulas:

(6) $$k=\frac{1}{2}\left(\bar{uu}-\bar{u}\bar{u}\right)$$

(7) $$\nu_{t}=k^{0.5}C_{t}V^{0.33}$$

where *C_t_*—constant; *V*—volume of the computational cell.

Gas phase injections were described using the discrete phase model (DPM) with stochastic behavior of the bubbles in the metal volume (Discrete Random Walk), described in detail in this work [47]. The discrete phase model included procedures of interaction with the continuous phase and updated the DPM source for every flow iteration.

(8) $$\frac{du_{b}}{dt}=\frac{18\mu C_{D}Re}{24\rho_{b}d_{b}^{2}}u-u_{b}+\frac{g\left(\rho_{b}-\rho \right)}{\rho_{b}}+\frac{1}{2}\frac{d\left(u-u_{b}\right)}{dt}$$

where *u_b_*—bubble velocity; *C_D_*—drag coefficient; *Re*—Reynolds number; *µ*—dynamic viscosity; *ρ_b_*—bubble density; *g*—gravitational acceleration.

The nitrogen volumetric flow rate was 2214 NL/min with an initial diameter of bubbles calculated by Equation (9) [65]. During numerical simulations, the flow rate was scaled by the face area of the nozzle in the lance tip. The numerical simulation took into account the gas expansion (Equation (10)), wherein it was homogeneous due to the homogenized temperature field [66].

(9) $$d_{b}=\frac{6\sigma d_{n}}{\rho g}$$

(10) $$d_{b_{1623}}=d_{b}{\left(\frac{\rho_{b_{300}}}{\rho_{b_{1623}}}\right)}^{0.33}$$

Taking into account the phenomenon of gas expansion in the numerical model was one of the necessary conditions to advance toward the industry process conditions [67]. The temperature of the liquid hot metal was 1623 K; the diameter of the gas bubbles after expansion was 0.0281 m and was constant. The numerical simulations did not take into account the process of bubble fusion and their growth resulting from mutual interactions; therefore, during the process of bubbles flowing through the hot metal, their diameter was kept constant. The nitrogen density at 1623 K was 0.21 kg/m^3^ calculated on the basis of the ideal gas law formula. The free surface of the liquid hot metal was described by a wall function with zero tangential stresses (0-x, 0-y, 0-z shear stress components), bearing in mind the simplification of the interaction of the gas column with the liquid hot metal—the slag phase interface [5,68]. Therefore, it must be noted that by using a flat free surface of hot metal, the influence of wave phenomena or swirl motions on the mixing potential is ignored during simulations. For the side wall and bottom of the ladle, wall conditions with a no-slip concept were used. Heat losses on the walls and bottom of the ladle and on the free surface were assumed to be −3000 W/m^2^ and −15,000 W/m^2^, respectively. These values were considered in the simulation, based on the Authors’ experiences in the modeling of industrial processes. For bubbles interacting with the lance surface and ladle refractory, reflective boundary conditions were used. For all bubbles interacting with the upper surface of the hot metal, escape boundary conditions were applied. A Green-Gauss cell-based method was used to compute the gradient. The SIMPLEC algorithm was used to describe the pressure and velocity fields. Bounded Central Differencing-type discretization was used for the momentum and kinetic energy equations. Second-order discretization was used for the remaining equations. For the considered numerical model and all solved equations, the controlled level of residues was at a level lower than 0.001. The grid of numerical models consists of an average of 550,000 tetrahedral elements in a type of Tgrid with skewness value below 0.81. The number of elements in the grid was determined in previous investigations, where for a 130-ton ladle grid, a size between 40,000 and 170,000 was considered and tested [69]. In this work, divergence in the liquid metal velocity was nearly completely limited for the grid, which consisted of above 100,000 elements. For the mixing phenomena and tracer evolution in the hot metal bulk, the species model without reaction was used. During the computer simulation, 56 kg of the tracer was introduced into the system, located at the moment of initiation of the mixing process under the top injection lance, in accordance with the tracer administration procedure adopted during laboratory experiments with the water physical model. The mixing process described by the results of numerical simulations and laboratory experiments on the water model was also verified by empirical correlations (11) and (12) describing the mixing time based on the potential mixing power [70,71]. Moreover, using empirical Equations (13) and (14) [70] relating to the constant rate of the desulfurization reaction resulting from the mixing dynamics of the heterogeneous system, the relationship between the hydrodynamics of the system and the thermodynamic parameters limiting sulfur transport processes between the phases occurring in the working volume of the ladle was assessed. The FactSage 7.2 computer program with the Fact database was used for the thermodynamic calculations.

(11) $$\varepsilon_{p}=14.23\frac{QT}{M}log\left(1+\frac{H_{i}}{1.48P_{0}}\right)$$

(12) $$t_{mix}=116{\left(\varepsilon_{p}\right)}^{-\frac{1}{3}}\left(D^{\frac{5}{3}}{H_{m}}^{-1}\right)$$

(13) $$\ln\frac{\%S_{0}-\%S_{eq}}{\%S-\%S_{eq}}=kt$$

(14) $$k=8\cdot 10^{-6}{\left(\varepsilon_{p}\right)}^{2.1}$$

where *t_mix_*—mixing time; *ε_p_*—mixing power; *T*—temperature; *M*—bath weight; *Q*—gas flow rate; *H_i_*—depth of gas injection; *H_m_*—depth of hot metal; *P*_0_—gas pressure at the bath free surface; *k*—rate constant; *D*—average diameter of ladle; *t*—time; *S*—sulfur content after treatment; *S*_0_—initial content of sulfur; *S_eq_*—sulfur content at equilibrium.

## 4. Results and Discussion

### 4.1. Hydrodynamic Model Validation

**Figure 3:**
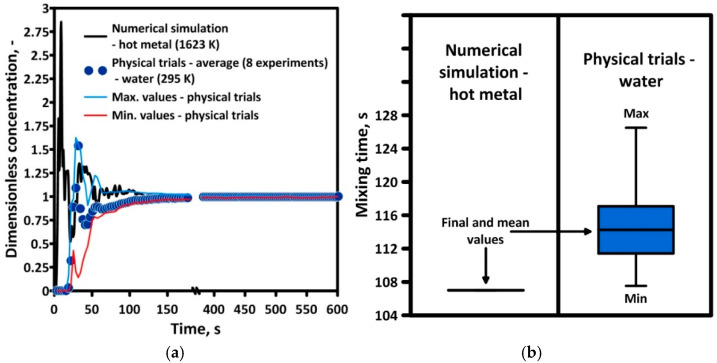
Validation of numerical results: (**a**) mixing curves, (**b**) mixing time.

Figure 3a shows the mixing curves for the considered period of pretreatment for Ladle No. 1. The tracer concentration (*C_mix_*) in the numerical and physical models was converted into dimensionless values according to Equation (15). The processing time from the physical model was converted into real-time according to Equation (2).

(15) $$C_{mix}=\frac{C_{t}-C_{0}}{C_{f}-C_{0}}$$

where *C_t_*—temporary concentration of tracer; *C*_0_—initial concentration of tracer; *C_f_*—final concentration of tracer.

The distribution of the mixing curve in the measurement zone is described by a peak and a tail with a sinusoidal distribution with a gradually flattening and disappearing amplitude. In order to quantitatively assess the efficiency of the numerical model, the mixing time was calculated, which was defined as the period of time necessary to obtain a 95% level of chemical homogenization. The mixing time of the tracer with the liquid hot metal was 107 s, while the average mixing time obtained during experiments on the water model was 114 s (Figure 3b). The mixing time obtained during laboratory experiments (water trials) was characterized by an almost 20-s difference between the minimum and maximum mixing time values. The standard deviation (σ) value amounts to 8 s. However, taking into account the linear scale of the physical model and the simplification of the numerical model, the description of the hydrodynamic phenomena in the ladle is comparable, especially since—based on the empirical equation of Mazumdar and Guthrie (12) [71]—the mixing time for the numerical model of Ladle No. 1 used for validation was 108 s, coinciding with the result obtained from the computer simulation. Moreover, the numerical model was checked by additional physical trials. During these cases, the top injection lance shifts from the center axis of the ladle near the ladle wall (one-third of the ladle’s bottom/top radius: 0.65 m). The off-center position of the gas injection in the bath reactor improves the mixing phenomena and decreases mixing time [72,73]. For Ladle No. 1, changing the top lance position closer to the ladle wall decreased the mixing time by 30%. Furthermore, the mixing time values obtained from numerical simulations are quite close to the values predicted by physical trials. The difference between the numerical and physical values of the mixing time amounts 5 s but with high values of standard deviation (Table 1).

### 4.2. Hot Metal Flow Structure in Ladle No. 2

**Figure 4:**
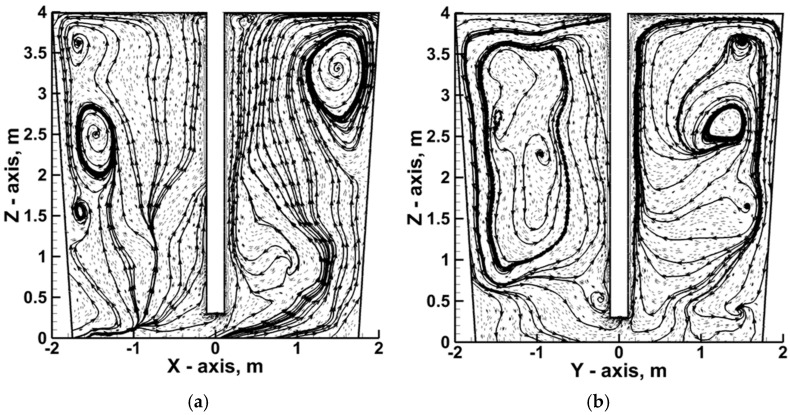
Hot metal streamlines in Ladle No. 2: (**a**) fluid flow behavior at plane Z–X; (**b**) fluid flow behavior at plane Z–Y.

Figure 4 shows the distribution of the streamlines on planes intersecting the axis of the ladle to illustrate the hydrodynamic structure formed in the ladle during nitrogen injection.

In the zone of influence of the injection lance, a decisive upward movement toward the free surface of the liquid metal is observed. Along the side walls of the ladle, the liquid metal falls toward the bottom, making the classic recirculation of liquid hot metal visible in a vertical arrangement. However, the hydrodynamic structure in the ladle is characterized by moderate asymmetry in relation to the axis of the top injection lance, visible by local metal circulations located approximately in the area of one-half and three-quarters of the height of the metal column.

**Figure 5:**
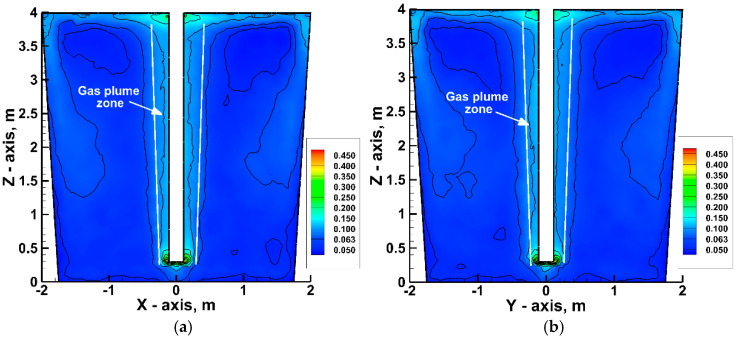
Relation between kinetic energy and velocity in the bulk of hot metal (Ladle No. 2): (**a**) turbulence intensity at plane Z–X; (**b**) turbulence intensity at plane Z–Y.

Figure 5 shows the distribution of the turbulence intensity of liquid hot metal described by Equation (16).

The turbulence intensity allows for the description of the gas plume zone created by injected gas. Generally, in the classical plume generated by the bottom plug, turbulence intensity is higher than 0.5. From the momentum and buoyancy to the surface region, a gas plume create the mixing phenomena inside the reactor [74,75]. Of course, in using the top lance, the core of the plume is dissipated around and along the lance, and the plume is repeatedly deformed. The zone of the highest turbulence intensity with a value of 0.45 is located at the top injection lance head. Moreover, an area of increased turbulence occurs along the lance and at the free surface surrounding the place where the injection lance is immersed in the liquid metal (average value of turbulence intensity in the plume, 0.1). This zone is directly correlated with the main flow stream and the stay of gas bubbles in the volume of liquid hot metal before they leave the heterogeneous system through the free surface. In the bulk of the hot metal turbulence, the intensity averages 0.05. Therefore, the best zone for intensively mixing compounds and their interaction occurs near the top of the lance.

(16) $$t_{i}=\frac{\sqrt{\frac{2}{3}k}}{u}$$

where *k*—turbulence kinetic energy; *u*—velocity.

**Figure 6:**
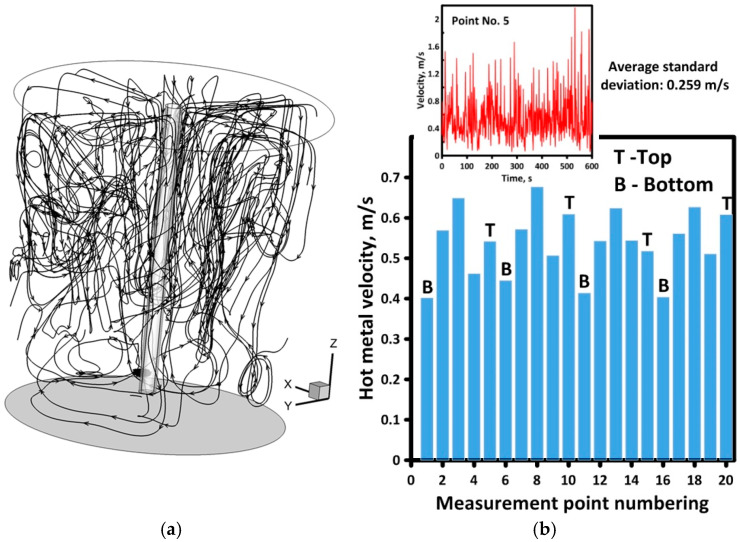
Hydrodynamic structure in the bulk of hot metal Ladle No. 2: (**a**) global flow paths; (**b**) velocity at measurement points.

In order to describe the hydrodynamic structure in a macroscopic context, flow path lines of the main streams of liquid hot metal in the working volume of the ladle were generated, presenting the hydrodynamic system in Figure 6a. Generally, vertical recirculation is observed in the ladle but locally horizontal recirculation is also observed, located mainly in the lower part of the batch reactor. Intensive blowing of the metal bath, additionally intensified by gas expansion, creates an unsteady hydrodynamic image in the tested object. The above statement is confirmed by the distribution of the velocity values of the liquid hot metal recorded at one of the measurement points (measurement point No. 5), which asymptotically assumes the maximum and minimum values with an average standard deviation of 0.259 m/s over a period of 10 min (Figure 6b).

**Figure 7:**
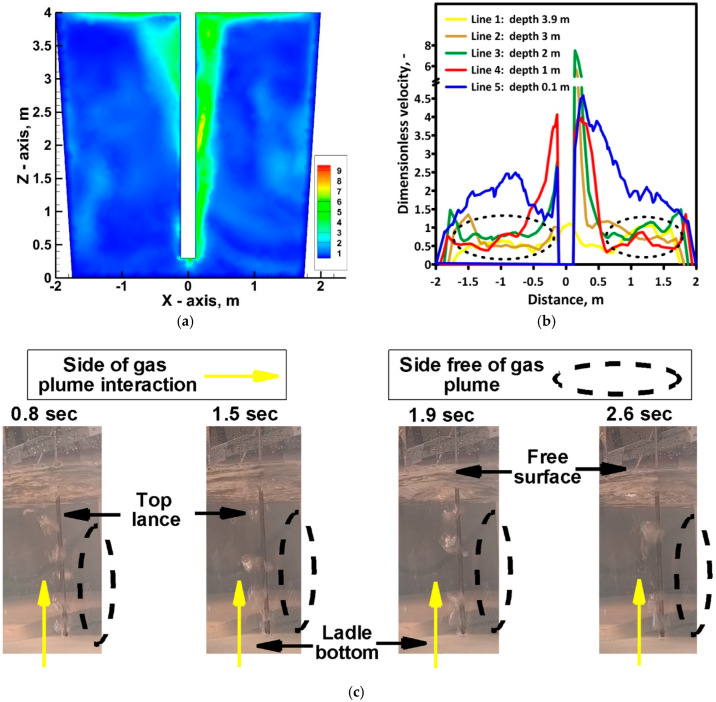
Top lance agitated ladle hydrodynamics: (**a**) planar view of dimensionless velocity distribution—numerical model; (**b**) dimensionless velocity distribution along measurement lines—numerical model; (**c**) behavior of gas plume—physical water model.

The velocity values obtained at the individual measurement points ensure dynamic flow in the working volume of the ladle, guaranteeing continuous transport of reagents to the potential reaction zone located in the injection lance zone and the free surface of the hot metal. Moreover, a lack of stagnation zones, which limit the mass transport phenomena, was confirmed by the relationship between the local velocity of hot metal and the average hot metal velocity in the bulk. The average value equals 0.591 m/s. Figure 7a presents the field of dimensionless velocity in the bulk of hot metal. Dimensionless velocity was calculated as the quotient of local velocity to average velocity. From the distribution of the dimensionless velocity along the lines at different depths of hot metal, we can see that the dimensionless velocity in the range between 0.5 and 1 occurred in the largest volume of metal bulk (Figure 7b). Moreover, values close to 5 in the zone of gas injection on the one side of the lance confirm the unsteady nature of the gas plume. This phenomenon was observed during physical trials (Figure 7c) and influenced the decision that the LES model would be more adequate for simulations of the considered system.

### 4.3. Mixing Phenomena

**Figure 8:**
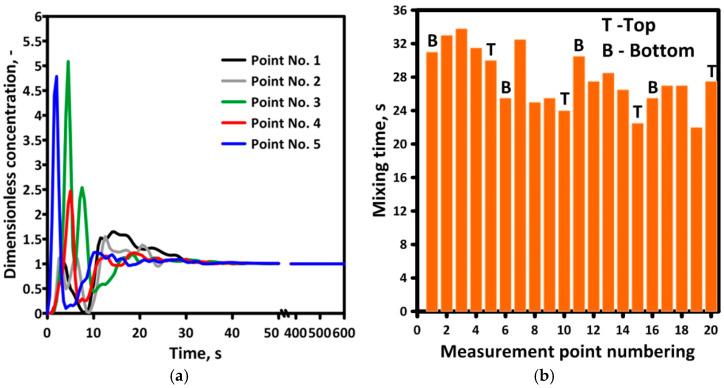
Numerical model of tracer behavior in the full-scale industry model of a ladle: (**a**) mixing curves; (**b**) mixing time.

In order to determine the mixing time for a 315-ton ladle (Ladle No. 2), a tracer was introduced into the system and the change in its concentration was recorded at measurement points 1–20 for 10 min. The distribution of the mixing curves indicates that within 10 s from the initiation of the tracer injection, its poor dispersion in the continuous medium is visible; however, over the next 20 s, the tracer concentration distribution normalizes, reaching the target state of 95% of the chemical homogenization level (Figure 8a).

Based on the mixing curves determined for each measurement point, the mixing time required to achieve 95% chemical homogenization was calculated. The lack of zones diagnosed in the reactor in question displays signs of stagnation areas, which could significantly slow down the mass transfer processes in the system under consideration and has been confirmed by the obtained mixing time values (Figure 8b).

### 4.4. Mass Transfer Controlled Rate—Influence of Process Parameters

**Figure 9:**
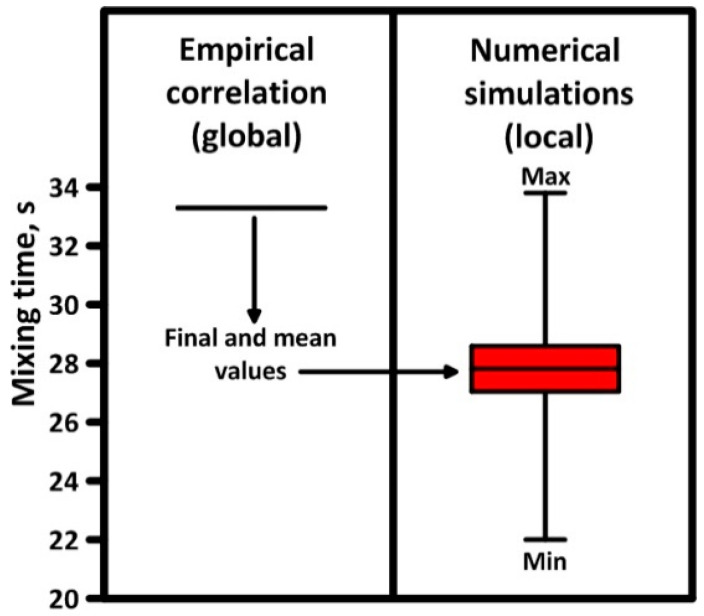
Mixing time for 300-ton ladle—validation of empirical Equation (12).

In order to verify the empirical Equations (11)–(14) with a numerical model, the mixing times obtained during computer simulations for Ladle No. 2 were compared with the time calculated on the basis of Equation (12). The average mixing time from the computer simulation was approximately 28 s, while the mixing time based on the empirical correlation was 33 s (Figure 9).

Obtaining similar mixing time values in this way, it was concluded that both the empirical correlation of Equation (12) and the simplified numerical model do not take into account the free surface ripples or the evolution of bubbles through their mutual interactions, describing turbulence using the large eddy approach (LES), which reliably describes the macromixing process in the ladle. Moreover, on the indicated differences in mixing time, the influence source that describes the rate mixing is global in Equation (12) (dimensions of ladle and mixing power) and local in the CFD model (velocity fluctuation and dissipation rate of turbulence kinetic energy). Therefore, by taking into account the phenomenon of gas expansion during nitrogen injection, the Authors calculated the real mixing power via Equation (11) for Ladle No. 2. Hence, the mixing energy for Ladle No. 2 was 478 W/t. Having also indirectly verified empirical Equation (11), Equation (14) based on the mixing energy was used, enabling the estimation of the rate constant k, which for Ladle No. 2 amounted to 3.39 min^−1^. The obtained value of the rate constant indicates very intensive conditions for the mass transport process, shaped by the nitrogen plume structure. However, taking into account the influence of not only the hydrodynamic parameters on the hot metal desulfurization process but also the thermodynamic interactions clearly resulting from the left side of Equation (13), an analysis of the thermodynamic state in the considered heterogeneous system was performed.

**Figure 10:**
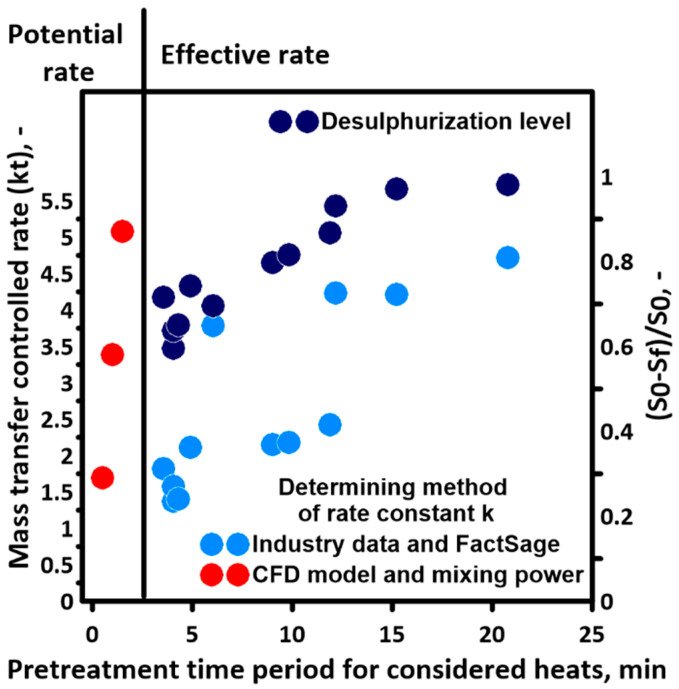
Potential and effective rates of hot metal desulphurization.

Research on the dynamics of mixing the metal bath during the hot metal desulfurization process is of fundamental importance for the description of the kinetics of desulfurization itself. The previously indicated relationships between mixing energy and the value of the desulfurization reaction rate constant are well-documented in the scientific literature. One example is the given Equation (14). However, taking the kinetic Equation (13) as a basis for further considerations, it should be noted that this description requires knowledge of the equilibrium state of the system under study. Therefore, the thermodynamic area (effective rate) presented in Figure 10 is diagnosed using numerical calculations.

For the purposes of the research, data sets were prepared for calculations in the FactSage 7.2 program. An attempt to capture the dependence of the equilibrium level on the main technological parameters of the process led to very interesting conclusions regarding the influence of nitrogen—as a gas transporting reagents to the ladle—on the equilibrium sulfur content in the metal. It should be noted that for low values of the nitrogen share, which are consistent with the amounts used in typical hot metal desulfurization installations, the effect of nitrogen on the final equilibrium sulfur content is the greatest. The nitrogen flow rate was converted into a percentage by weight of the system, and the equilibrium state was determined in a loop for a variable nitrogen share every 0.1%. The consequence of the observation was a modification of the assumptions for equilibrium calculations based on industrial research. The most important change in the approach to calculations was the abandonment of the search for balance for the entire mass of metal in the ladle related to the total volume of nitrogen used. It was decided that the level of equilibrium sulfur content [S]_eq_ appearing in Equation (13) will be calculated in relation to the mass of hot metal that is in direct contact with nitrogen bubbles and was estimated by FactSage 7.2 software. Theoretical determination of the value of the mentioned mass is difficult and can be treated as a parameter allowing for tuning the model to real conditions. The conclusion is that the nitrogen introduced into the system is not only of purely mechanical importance as a source of energy for mixing the bath but the volume of nitrogen introduced affects the thermodynamic barriers of the process. In order to determine the range of variability in the desulfurization reaction rate constant from Equation (13), simulations were carried out for typical cases that may be encountered during a real industrial process. This variation included both the desulfurization time, temperature, and weight of the injected desulfurization agents. The data for calculations of k are summarized in Table 2.

It is important that data on the initial and final sulphur content, temperature process, and time of treatment were collected during industry trials. The obtained results indicate that the value of the reaction rate constant for the desulfurization of hot metal obtained based on the analysis of process data ranges from 0.2 to 0.6 min^−1^. This value is much lower than that resulting from Equation (14). Mathematically, it is possible for the values of k to be close to those obtained from Equation (14) but then the condition that [S]_eq_ is almost equal to [S] from Equation (13) should be met. The aim of this research was to check the correctness of the predictions of kinetic Equation (13) in two different ways. In the first approach, the mixing power values obtained from the hydrodynamic CFD model were taken as the basis. In this case, it was assumed that the results obtained for the cold model allow us to assume the correctness of the forecast in relation to the actual hot system. The second approach used sample data from hot metal desulfurization processes, Table 1. These data provided all the quantities necessary in Equation (13), except the rate constant k and the value of the equilibrium sulfur content S_eq_; however, the S_eq_ value can be determined on a case-by-case basis using the Equilib module of FactSage 7.2 software. Figure 9 shows the results describing the mass transfer controlled rate (kt) for the desulfurization process based on Equation (13), taking into account rate constants determined on the basis of the mixing power (CFD model and empirical Equation (14)) and the thermodynamic components’ interaction from real high-temperature systems (Equation (13)). It should be emphasized that each point on the graph represents a separate heat (process) and, therefore, Figure 9 is only an approximate measure of the trend that can be observed during desulfurization.

In addition, the values of the rate constant and pretreatment time of heat indicate that the process efficiency greatly changes. This fact confirms the values of the sulphur removal degree in the range of 0.6 to 0.98. The obtained results clearly indicate that the desulfurization process of hot metal is determined by both hydrodynamic (reactor dimensions, position of the injection lance, and working gas flow rate) and thermodynamic (concentrations of components of co-existing phases, temperature, and pressure) areas. Therefore, intensive mixing without the required amount and chemical compositions of the desulphurization agents and slag phase does not allow for obtaining highly effective levels of sulphur removal. Hence, a description of the desulphurization process by Equation (14) alone is too much of a simplification. The red points in Figure 9 indicate adequate mixing conditions in Ladle No. 2. According to Equation (14), in the 315-ton ladle, after one minute of strongly agitated hot metal by nitrogen plume, the required value of mass transfer controlled rate is obtained (potential rate area). However, based on real industry system data at a similar value of the mass transfer controlled rate, one minute of the pretreatment process is not sufficient. When pretreatment occurs for longer than 10 min, the degree of the sulphur removal gain level is above 0.9.

## 5. Conclusions

Based on laboratory experiments with a water model, numerical computer simulations, and industrial observations, the following was found:

1. Physical experiments confirmed the applicability of using a simple LES numerical model for predictions of macromixing conditions for the considered system;
2. The hydrodynamic conditions diagnosed in the 315-ton ladle with intensive nitrogen injection of 2.2 Nm^3^/min indicate the absence of stagnation areas, which is significant from the point of view of mixing kinetics. This fact is confirmed by the approximately 30 s of mixing time for 95% chemical homogenization diagnosed in different areas of the reactor working volume;
3. Verification of the empirical relationships (11,12) by numerical and physical model tests showed that the description of the macromixing process should take into account the phenomenon of gas expansion occurring in real industrial conditions when a high-temperature system is considered, in accordance with previous investigations of other research groups;
4. Obtaining a high value of the rate constant determined on the basis of only the hydrodynamic area, based on mixing power, does not guarantee obtaining low sulfur content because the success of the desulfurization process is also determined by the thermodynamic area. The relationship k as a function of mixing power (Equation (14)), known from previous studies [70], requires control of its applicability, and the criterion should be the distance of the system from the equilibrium state after the process is completed. In other words, the applicability of the rate constant based only on mixing power is not sufficient to predict effective sulfur removal during hot metal pretreatment processes, in accordance with presented industry trial results;
5. Based on the data from the high-temperature system, it was found that the rate constant of the desulfurization process varies within a wide range, affecting the efficiency of the sulphur removal degree from values 0.6 to 0.98;
6. In the future, the Authors plan to develop a numerical model to simultaneously calculate the hydrodynamics and chemical reaction rate phenomena between hot metal components and desulphurization agents.

## Figures and Tables

**Table 1:** Model validation for off-center location of top injection lance in Ladle No. 1.

**Case**	**t_mix_**				**σ**
	**Numerical Model**	**Physical Experiments**			
		**Ave**	**Min**	**Max**	14
1/3 of radius	73	78	60	91	

**Table 2:** Sulfur content in the hot metal during industry pretreatment process and values of rate constant.

Case No.	S_0_, [wt. %]	S, [wt. %]	S_eq_, [wt. %]	Temperature, [K]	Time, [min]	k, [min^−1^]
1	0.0517	0.001	0.0005	1648	20.7	0.227
2	0.0328	0.001	0.0005	1656	15.2	0.277
3	0.0287	0.002	0.0016	1606	12.1	0.347
4	0.0528	0.007	0.0025	1643	11.8	0.203
5	0.0246	0.007	0.0035	1645	3.56	0.509
6	0.0433	0.008	0.0035	1632	9.81	0.222
7	0.0395	0.008	0.0038	1631	9.01	0.238
8	0.0310	0.008	0.0048	1656	4.90	0.430
9	0.0344	0.012	0.0046	1649	4.30	0.325
10	0.0395	0.012	0.0113	1647	6.05	0.625
11	0.0370	0.015	0.0075	1644	4.05	0.338
12	0.0416	0.015	0.0082	1639	4.06	0.388

## Data Availability

The data are contained within the article. Raw data that support the findings of this study are available from the Authors upon request.
